# Long-Term Results of Thoraco-Pleuro-Pneumonectomy (TPP) for Recurrent Thoracic Sarcomas

**DOI:** 10.1245/s10434-017-6219-2

**Published:** 2017-11-20

**Authors:** U. Pastorino, P. Scanagatta, P. Girotti, L. Rolli, A. Gronchi

**Affiliations:** Department of Surgery, Fondazione IRCCS Istituto Nazionale dei Tumori, Milan, Italy

## To the Editors:

In 2014, we published a series of four consecutive cases of thoraco-pleuro-pneumonectomy (TPP) with en bloc resection of the entire lung, chest wall and diaphragm, and immediate rib-like reconstruction for recurrent thoracic sarcomas. The aim of surgery was to maximize the chance of long-term tumor control and possibly cure. We report here the long-term results of this extreme procedure, analyzing the oncologic follow-up of patients, along with the long-term functional and morphological changes of the rigid reconstruction using the rib-like technique. The four patients are alive and free from thoracic relapse after 69, 65, 64, and 54 months following TPP. The fourth patient, with Ewing’s Sarcoma, underwent chemotherapy, radiotherapy, and subsequent left nephrectomy for an extrathoracic metastasis that occurred 17 months after surgery. The long-term features of chest wall reconstruction show progressive changes in the shape and functionality of the three-dimensional, rib-like prosthesis. In selected patients with advanced low- to intermediate-grade recurrent thoracic sarcomas, one-stage TPP provides long-term tumor control. Prosthetic replacement requires substantial improvements to maintain the shape and functional profile of the artificial chest wall in the long run.

*Introduction* Radical resection is essential to cure thoracic sarcomas, but is often technically demanding and difficult to achieve. In 2014, we published our single-center institutional experience of surgery for recurrent thoracic low- to intermediate-grade sarcomas, describing the surgical technique of one-stage thoraco-pleuro-pneumonectomy (TPP) and immediate rigid prosthetic rib-like reconstruction of the chest wall.^1^ Herein, we analyze the long-term results of the four patients operated from an oncologic and functional/anatomical point of view.

*Methods* TPP is the removal of the entire hemithorax (from the first to twelfth ribs), lung, and diaphragm, preserving the major thoracic muscles. Four consecutive patients with recurrent thoracic sarcoma were selected and underwent TPP at our institution from November 2011 to January 2013.

Patient selection, surgical approach, TPP technique, and the following one-stage reconstruction ‘rib-like’ procedure have been fully described in our previous article,^1^ and summarized for readers in Tables 1 and 2 of the electronic supplementary material. All patients underwent a postoperative basal chest computed tomography (CT) scan prior to discharge, and were subsequently followed up by our surgical oncologic outpatient service through sequential repetition of chest CT scan or magnetic resonance imaging (MRI) according to the case-by-case clinical findings.

**Fig. 1:**
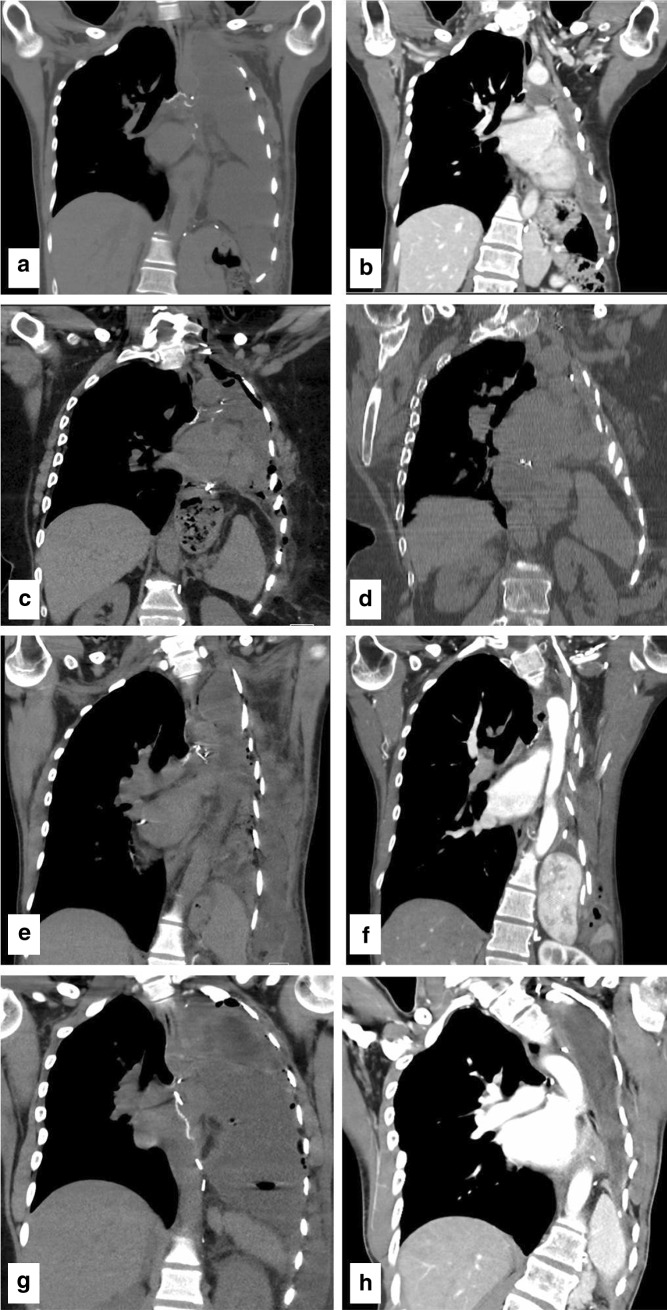
Postoperative coronal view of a chest computed tomography scan at the carinal bifurcation/left bronchial stump level. Patient 1: (**a**) 14th postoperative day and (**b**) 66 months after surgery. Patient 2: (**c**) 27th postoperative day and (**d**) 62 months after. Patient 3: (**e**) 9th postoperative day and (**f**) 60 months after. Patient 4: (**g**) 15th postoperative day and (**h**) 54 months after

*Results* Patient characteristics and outcomes are summarized and updated in Table 3 in Supplementary material. The four patients are alive and free from thoracic relapse after 69, 65, 64, and 54 months following TPP, respectively. The fourth patient, a male adolescent affected by Ewing’s Sarcoma, underwent chemotherapy, radiotherapy, and subsequent surgery for an abdominal metastasis, which occurred 17 months after surgery. The long-term results of the rib-like reconstruction of the chest were evaluated through clinical observation of the patients and analysis of chest CT scan findings (Fig. 1). All patients developed a change in the shape and external appearance of the reconstructed hemithorax, and a worsening left-convex scoliosis, which was more evident in patients 2 and 4 (Fig. 1c, d, g, h). In the third patient, we observed a progressive approximation of the prosthesis to the mediastinum (Fig. 1e, f). Nonetheless, there were no reports of chronic chest pain, upper girdle dysfunction, shortness of breath on moderate exercise, or digestive disorders. In particular, all patients were able to attend to their daily activities and three (patients 1, 3 and 4) kept their active job.

*Discussion* Surgery is the treatment of choice for localized sarcoma whenever a complete resection of the tumor can be obtained, with adequate cuff of surrounding healthy tissue. This may imply resection of surrounding viscera, even if not macroscopically involved.^2^^–^ 7 In patients affected by recurrent thoracic sarcomas, such resections may require extended chest wall, lung, or major vessel resections to avoid or minimize the risk of tumor cells at the surgical margins.^8^^–^ 11

In their invited commentary on our article, Bauer and Berkhelm outlined the importance of proper reconstruction of the involved hemithorax in order to reflect the characteristics of normal chest physiology.^12^ The ‘rib-like’ technique was described as an efficient solution for reconstructing large thoracic defects after resection of sternal tumors,^13^ or in association with diaphragmatic resections,^14^ fulfilling the four ideal Le Roux characteristics of a prosthesis: rigidity, malleability, radiolucency and inertness.^15^ In addition, this technique guaranteed a prolonged postoperative permeability to body fluid and cells, thereby avoiding the risk of prosthetic infection, with the consequent need for removal.

Long-term follow-up data confirm the oncological rationale of TPP in selected patients affected by advanced recurrent thoracic sarcomas: the four patients in our series are locally tumor-free, and only one underwent further treatment for a distant relapse. This evidence confirms that one-stage TPP is feasible and safe, and could lead to long-term tumor control.

On the other hand, as clearly shown in Fig. 1, stability and durability of thoracic prosthetic reconstruction should be improved, along with its functional and aesthetic long-term outcomes.

The handmade, intraoperative molding of the rib-like prosthesis did not guarantee the required uniformity, consistency, and permanent geometric stability of such an extensive chest wall replacement. This is an important open issue, but the goal could be achieved by engineering development, combined with new biocompatible materials able to balance rigidity, flexibility, and inertness. In fact, the artificial prosthesis should durably mimic the shape and function of the native chest wall, without increasing the risk of infection. More recent development of this experience has enabled us to produce a custom-made industrial prosthesis, which is presently under clinical testing.

While TPP has proved effective in achieving local control and long-term survival, we need better technology to improve the quality of life of these patients.

## Electronic supplementary material

Below is the link to the electronic supplementary material.

Supplementary material 1 (DOCX 14 kb)
