# Supplementary material for: Long-Term Results of Thoraco-Pleuro-Pneumonectomy (TPP) for Recurrent Thoracic Sarcomas
Source: Ann Surg Oncol. 2017 Nov 20;24(Suppl 3):551–4. doi: 10.1245/s10434-017-6219-2 (PMC5721124; doi:10.1245/s10434-017-6219-2)
Supplement: Supplementary file 1 — Supplementary material 1 (DOCX 14 kb) [file 10434_2017_6219_MOESM1_ESM.docx]

**TABLE 1:** Patient Selection Checklist: all this clinical criteria should be present. FEV1 Forced Expiratory Volume in 1 second.

| Preoperative pathologic confirmation of intrathoracic sarcoma |
| --- |
| Disease limited to one hemithorax |
| Pulmonary involvement requiring pneumonectomy |
| Diaphragmatic and chest wall (>3 ribs) involvement |
| Age ≤ 70 years |
| Postoperative predicted FEV1 and carbon monoxide diffusing capacity > 40% |
| American Society of Anesthesiology (ASA) score class I or II |
| No previous cardiac surgery |
| Multidisciplinary Sarcoma Tumor Board Discussion |
| Informed consent signed |

**TABLE 2:** Summary of the surgical steps of one-stage thoracopleuropneumonectomy and riblike reconstruction. * Surgimesh, Aspide Medical, La Talauderie, France ** Mendec Cranio, Tecres Medical, Sommacampagna, Italy; Cranioplastic Type 1-Slow Set, DePuy International Ltd., Blackpool, UK

| Position (decubitus) | Posterolateral |
| --- | --- |
| Incision | L shaped (from 7th cervical vertebra to second lumbar vertebra, then following 12^th^ rib) |
| Myocutaneous flap preparation and lifting together with the scapula | Inferoposterior insertion of latissimus dorsi, tapezius and rhomboid muscle sectioned; anterior insertions on the ribs sectioned, spinal muscles are dissected downward from each transverse process |
| Thoracopleuropneumonectomy | Rib disarticulation (from I to XII)  Intercostal pedicles ligation and section  Isolation and section pulmonary artery, main bronchus and veins  Anterior rib section (from I to the costal arch)  Diaphragmatic resection (from the medial margin) |
| Riblike reconstruction with 3D chest wall prosthesis | Omentum is pulled upward and fixed to pericardium and posterior abdominal wall  Polyethylene multifilament knitted mesh* and two resins** are modeled on a chest aluminium cast  A free portion of mesh (20 cm) is used to reconstruct diaphragm  Riblike prosthesis is fixed to the sternum and transverse processes; the free mesh portion is fixed to diaphragmatic remnants and pericardial margin  Pectoralis, serratus and long posterior muscles are anchored to the “intercostal” mesh component  Myocutaneous flap is repositioned to cover the prosthesis |

**TABLE 3:** Patient characteristics (for further details see ref.1, Table 1) and long term follow-up. LGFMS low-grade fibromyxoid sarcoma, MSFT malignant solitary fibrous tumor, MPNST malignant peripheral nerve sheath tumor, ES Ewing sarcoma. NED no evidence of disease, AWD alive with disease, * see description in the text

| Variable | Patient 1 | Patient 2 | Patient 3 | Patient 4 |
| --- | --- | --- | --- | --- |
| Sex | Female | Female | Female | Male |
| Age (years) | 32 | 58 | 29 | 16 |
| Histology | LGFMS | MSFT | MPNST | ES |
| Residual viable tumor (%) | 90 | 90 | 90 | 80 |
| Mitotic Index (per 10 HPF) | 1 | 6 | 20 | 6 |
| Maximum tumor size (cm) | 12 | 18 | 14 | 14 |
| Follow-up (months) | 69 | 65 | 64 | 54 |
| Status | NED | NED | NED | AWD (thorax NED)* |
